# Supplementary material for: The causal role of circulating inflammatory markers in osteoporosis: a bidirectional Mendelian randomized study
Source: Front Immunol. 2024 Jul 18;15:1412298. doi: 10.3389/fimmu.2024.1412298 (PMC11291241; doi:10.3389/fimmu.2024.1412298)
Supplement: Supplementary file 1 [file DataSheet_1.zip › supplementary material/CIM-OSTEOPOROSIS /GCST90274806.csv_leave_one_out.pdf]

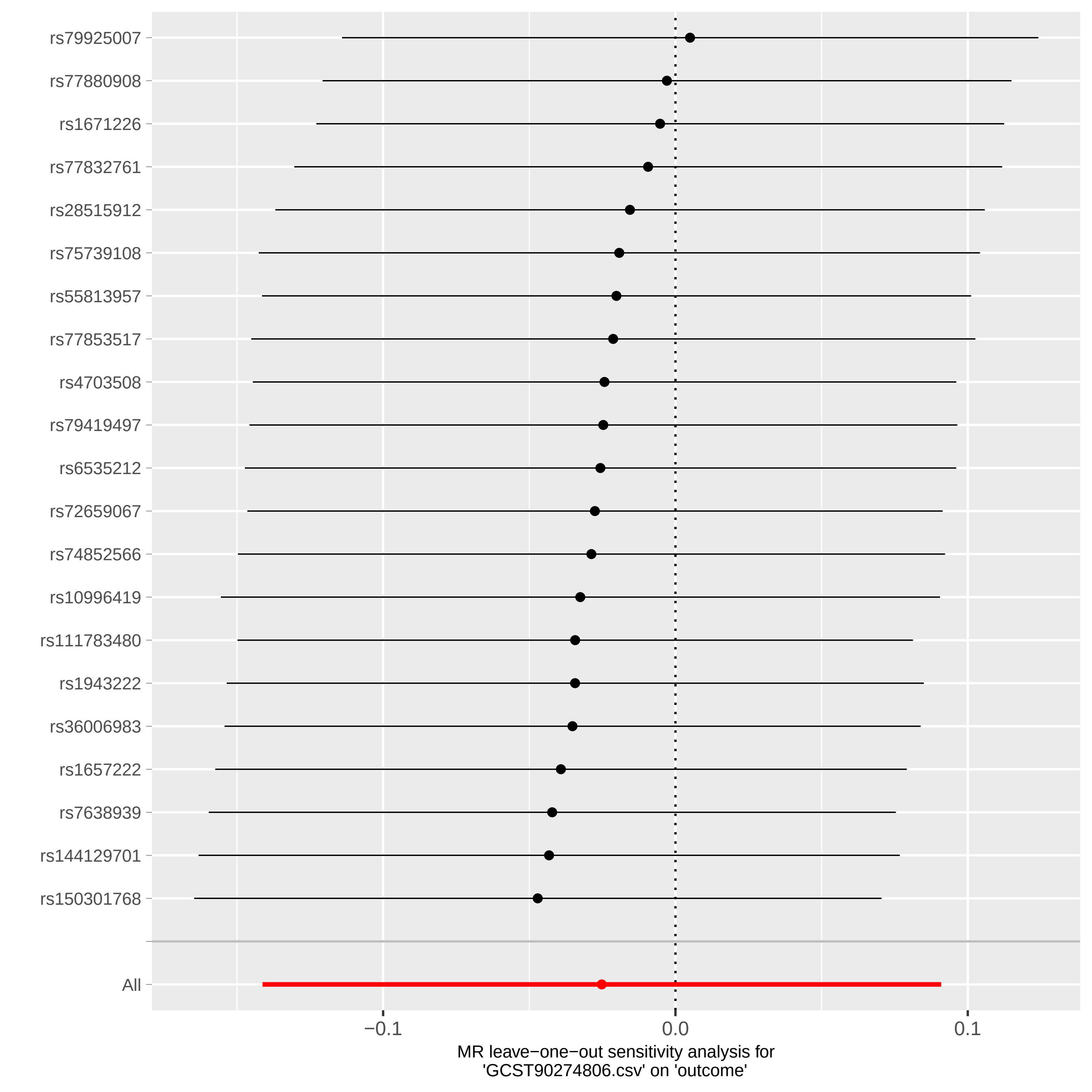
rs79925007
rs77880908
rs1671226
rs77832761
rs28515912
rs75739108
rs55813957
rs77853517
rs4703508
rs79419497
rs6535212
rs72659067
rs74852566
rs10996419
rs111783480
rs1943222
rs36006983
rs1657222
rs7638939
rs144129701
rs150301768
All
−0.1
0.0
0.1
MR leave−one−out sensitivity analysis for
'GCST90274806.csv' on 'outcome'
